# Managing opioid withdrawal precipitated by buprenorphine with buprenorphine

**DOI:** 10.1111/dar.13228

**Published:** 2021-01-21

**Authors:** Bridget Oakley, Hester Wilson, Victoria Hayes, Nicholas Lintzeris

**Affiliations:** ^1^ Drug and Alcohol Services South East Sydney Local Health District Sydney Australia; ^2^ School of Public Health and Community Medicine UNSW Sydney Sydney Australia; ^3^ Division Addiction Medicine University of Sydney Sydney Australia

**Keywords:** buprenorphine, precipitated withdrawal, treatment, opioid dependence, opioid withdrawal

## Abstract

Buprenorphine is a partial opioid agonist commonly used to treat opioid dependence. The pharmacology of buprenorphine increases the risk of a precipitated opioid withdrawal when commencing patients on buprenorphine treatment, particularly when transferring from long acting opioids (e.g. methadone). There is little documented experience regarding the management of precipitated withdrawal. In our case, a patient developed a significant precipitated opioid withdrawal following buprenorphine administration, and was able to be successfully treated in hospital with further buprenorphine. This demonstrates that rapid increases in buprenorphine dose can be used as an effective treatment for buprenorphine‐induced precipitated opioid withdrawal. The use of buprenorphine to manage withdrawal then allows the individual to continue on this highly effective treatment.

## Background

Buprenorphine/naloxone (bup/nx) combination is commonly used in Australia and international countries to treat opioid dependence, either from illicit opioids (heroin) or from pharmaceutical opioids (codeine, fentanyl, oxycodone, etc.) [1]. Like methadone, it is an evidence‐based, effective treatment for opioid dependence [2].

Buprenorphine is a partial agonist acting at the mu‐opioid receptor with high receptor affinity. In an opioid‐dependent person, buprenorphine can displace opioid agonists of lower receptor affinity (e.g. heroin, methadone) from the receptors, without full activation (partial agonism), leading to a ‘precipitated’ opioid withdrawal [3, 4].

Precipitated opioid withdrawal is characterised by the rapid onset of opioid withdrawal symptoms (such as aches, nausea and vomiting, diarrhoea and abdominal cramps, dilated pupils, running nose, yawning) within 1–2 h following the first dose of buprenorphine, and gradually subsiding over the subsequent 6–24 h. This is a well‐recognised adverse outcome from buprenorphine induction [3, 4, 5], and was found to occur in 9% of buprenorphine inductions [6]. Risk factors for precipitated withdrawal include transferring from long‐acting agents such as methadone, recent benzodiazepine use, no past patient experience with buprenorphine, and a low initial dose of buprenorphine/naloxone [6].

The Clinical Opiate Withdrawal Scale (COWS) is a tool that can be used to guide the diagnosis of an established opioid withdrawal [7]. Once the patient is in opioid withdrawal, buprenorphine can be started with a decreased risk of precipitating a withdrawal [4]. Despite the use of clinical monitoring tools like the COWS, precipitated withdrawal can still occur, as demonstrated in our case.

There are three potential responses to severe precipitated withdrawal: (i) reassurance and symptomatic medication (e.g. anti‐emetics, anti‐inflammatories, sedatives); (ii) adding further buprenorphine to increase opioid agonist effect; or (iii) abandoning buprenorphine treatment and reverting to treatment with full opioid agonists, such as methadone. Clinical experience suggests that once a patient has experienced precipitated withdrawal they are understandably reluctant to continue taking the medication that caused their distress. A case study in 2012 [8] described precipitated withdrawal in a patient on a daily dose of 165 mg of methadone with prolonged QTc (corrected QT interval), who was prematurely administered 4 mg of buprenorphine prior to onset of opioid withdrawal, resulting in significant withdrawal symptoms. This patient was successfully treated with buprenorphine in the intensive care setting, where he was given an increasing dose of up to 8 mg per day. In this case, the symptoms settled by day 4 on a dose of 32 mg [8]. Current Australian guidelines suggest further buprenorphine doses, however, they do not provide details on a dosing regimen [1, 5]. There are few case reports or studies to support this practice. This potentially results in patients discontinuing an evidence‐based treatment and increases the chance of return to illicit opioid use with inherent risks of harm.

## Case Study

A 53‐year‐old male patient with opioid dependence (as defined by the International Classification of Diseases, 10th Revision) presented to our inner‐city drug and alcohol service in Sydney, Australia. A long‐term user of illicit opioids, he had increasing intravenous heroin use, $AUD50 (approximately 0.1 g) each morning, with recent escalation to evening use as well.

He first used heroin aged 21 years, and his first episode of methadone treatment was at the age of 28 years. He was in treatment for 5 years and was able to reduce off methadone and did not use opioids for the following 10 years. Following the significant personal loss of his partner, he abused cocaine and became homeless, and returned to illicit methadone use in this context. In 2015, he developed an epidural abscess and staphylococcal septicaemia requiring intensive care admission. He continued to have chronic back pain after this and was prescribed opioids, including transdermal buprenorphine, to manage this. He was able to reduce and cease transdermal buprenorphine but relapsed to heroin use.

He was willing to commence treatment with sublingual bup/nx combination to cease heroin use. There was a history of daily tobacco smoking, no current regular medications, no alcohol or current other drug use, and no comorbid psychiatric history. He was working full time, had recently secured stable accommodation and lived alone. He had attempted treatment with sublingual bup/nx at a community pharmacy but had used heroin prior to this and suffered mild opioid withdrawal symptoms, and did not continue with treatment at this time. He had a comprehensive assessment at a specialist public drug and alcohol facility. He was educated about bup/nx treatment, including risks of precipitated withdrawal in the setting of methadone use, and given the opportunity to ask questions, as per the service's usual practice. He denied any current or recent methadone use at this time.

He presented to the specialist service for buprenorphine induction at 0900 h, however, he reported using just under $AUD50 heroin intravenously at 0300 h that morning and had no symptoms or signs of withdrawal. He was advised to return the following day and to abstain from further opioid use.

**Figure 1:**
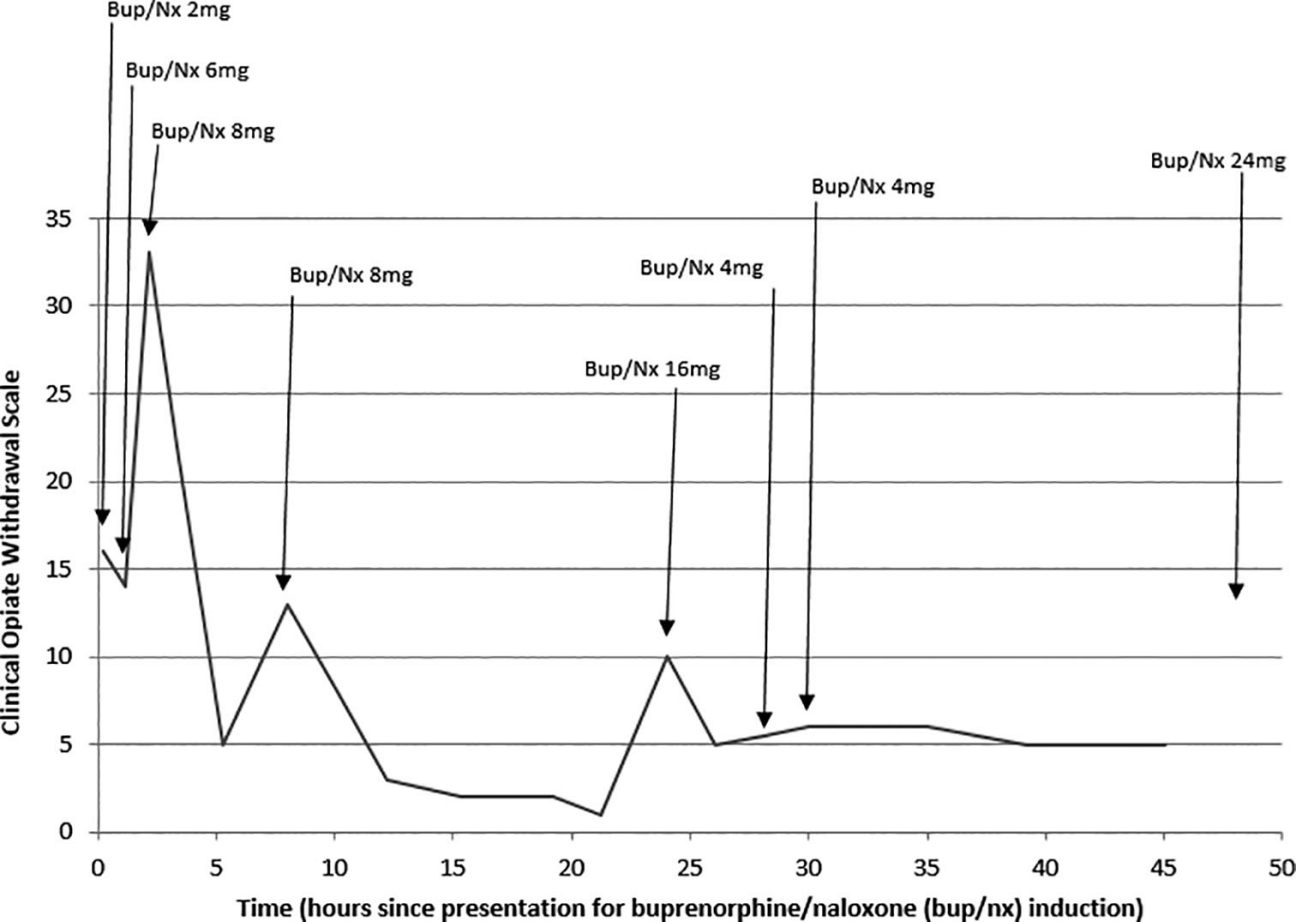
Timeline of Clinical Opiate Withdrawal Scale scores and buprenorphine/naloxone (bup/nx) dosing during inpatient admission.

On presentation to our clinic at 0800 h the following day he reported nil opioid use since taking heroin at 0300 h the previous day (29 h previously). He did not report taking any other opioids. He had a COWS of 16 (indicating moderate severity withdrawal) at 0820 h with signs and symptoms including nausea, piloerection, tremor, irritability, nasal stuffiness and dilated pupils (see Table 1). He was given a ‘test dose’ of 2 mg bup/nx combination sublingually (and all further doses given were sublingual). At 0914 h (1 h later), his COWS had decreased to 14 (see Figure 1). He was given a further 6 mg bup/nx combination to total 8 mg as an initial day 1 dose [1]. He reported soon after the second dose that he was feeling unwell. At 1020 h (2 h after first dose), his COWS had more than doubled to 33 with signs and symptoms including tachycardia, vomiting and diarrhoea, piloerection, dilated pupils, marked tremor, nasal stuffiness, bone and joint aches, extreme irritability and agitation. A precipitated opioid withdrawal was diagnosed—based on accepted definition of an increase in COWS of 6 or more within 2 h of initiating buprenorphine treatment [4]. Discussions regarding management options were held between the treating team, his prescriber and the patient. He had a strong therapeutic alliance with his prescriber who provided nuanced encouragement and support, and as a result he agreed to an additional 8 mg bup/nx combination given at 1020 h and was transferred to hospital for inpatient admission under the Addiction Medicine team. On arrival to the hospital, he suffered significant symptoms including anxiety and irritability, which was treated with a low dose sedative. Anti‐emetics and antispasmodics were also given on arrival for symptom management.

**Table 1:** Timeline of Clinical Opiate Withdrawal Scale (COWS) scores and treatment during inpatient admission

Time		COWS	Treatment	Comments
Day 1	0819	16	Bup/nx 2 mg SL	Test dose given when COWS >8 as per guidelines [9]
	0914	14	Bup/nx 6 mg SL	No increase in COWS, so further bup/nx given to make a total starting dose of 8 mg
	1019	33	Bup/nx 8 mg SL	Precipitated withdrawal diagnosed Transferred from community facility to hospital emergency department
	1135	22		Assessed in emergency department
	1203		Sodium chloride 0.9% 1L IV	Admitted Dehydration
			Ondansetron 4 mg	Nausea and vomiting
			IV Diazepam 5 mg PO	Agitation
			Buscopan 20 mg IV	Abdominal pain
	1327	5		Reduced COWS in response to greater bup/nx dose
	1600	13	Bup/nx 8 mg SL	Withdrawal symptoms
			Paracetamol 1 g PO	Pain
	1807	8	Ondansetron 4 mg PO	Nausea and vomiting
	1851		Paracetamol 1 g PO	Pain
	2023	3	Metoclopramide 10 mg IM	Nausea and vomiting
	2328	2	Paracetamol 1 g PO	Pain
Day 2	0115	2		
	0321	2		
	0523	1	Paracetamol 1 g PO	Pain
	0803	10	Bup/nx 16 mg SL	Rising withdrawal symptoms as time from last bup/nx dose increases 16 mg given rather than 24 mg (total day 1 dose) to allow monitoring for sedation, in a setting where further doses can be given if needed
			Ondansetron 4 mg PO	Nausea and vomiting
	1007	5	Paracetamol 1 g PO	Pain
	1215		Bup/nx 4 mg SL	Withdrawal symptoms not improving
	1401	6	Bup/nx 4 mg SL	Doses split to reduce risk of sedation
	1656		Paracetamol 1 g PO	Pain
	1903	6		
	2219		Ondansetron 4 mg PO	Prevention nausea and vomiting
	2257		Paracetamol 1 g PO	Pain
			Oxazepam 15 mg PO	Insomnia
	2321	5		
Day 3	0503	5		
	0605		Paracetamol 1 g PO	Pain
	0737		Ondansetron 4 mg PO	Prevention nausea and vomiting
	0800		Bup/nx 24 mg SL	COWS stable and scoring ceased 24 mg given as total daily dose on day 1 and 2
	1859		Paracetamol 1 g PO	Pain
	2212		Ondansetron 4 mg PO	Nausea and vomiting
	2247		Oxazepam 15 mg PO	Insomnia
Day 4	0800		Bup/nx 24 mg SL	Symptoms of precipitated withdrawal resolved
			Ondansetron 4 mg PO	Prevention nausea and vomiting Discharged for community management

Bup/nx, buprenorphine/naloxone; IM, intramuscularly; IV, intravenously; PO, by mouth; SL, sublingual.

Following the additional 8 mg bup/nx combination his COWS reduced to 22 at 1135 h and 5 at 1327 h. At 1600 h (8 h post‐initial bup/nx), COWS increased to 13, and he was given another 8 mg bup/nx combination. This brought his total day 1 dose to 24 mg. By 2000 h his COWS had settled to 3 and remained at 1–2 overnight. At 1600 h, he was noted to have a temperature of 37.9°C. This rose to 38.4°C with productive cough. These were investigated with a septic screen (chest X‐ray, blood cultures, full blood count, urine culture). No specific aetiology was found and there was no recurrence of fever throughout the remainder of the admission. While the symptoms of opiate withdrawal and febrile illness can appear similar, it is notable that the patient's symptoms of opiate withdrawal were minimal when the fever was present, as measured by a COWS of 13 decreasing to 3.

At 0800 h the following day his COWS was 10 prior to dosing, and he was given a morning dose of 16 mg bup/nx combination. His COWS dropped to 5–6 after this dose. An additional 8 mg was given in between 1200 and 1400 h and his symptoms settled, and remained so. The following morning 24 mg bup/nx combination was given and COWS monitoring ceased, as he reported he now felt comfortable on this dose, and there was no evidence of over‐sedation. He continued on 24 mg bup/nx combination and was able to be discharged on this dose. He continues to be in active treatment and has returned to work.

In our clinic, urine drug screens are performed at assessment and various times during treatment, however, they are not performed as a tool to guide induction because results take 3–7 days to return and so are unable to assist at the point of care. It was performed in this situation to assist with identifying a cause for the precipitated withdrawal. Urine was collected during inpatient admission and results available after discharge confirmed the presence of methadone and codeine. No additional opiates were identified. The patient had been prescribed methadone some years ago as a treatment for opiate dependence. On discharge review, he reported last taking a 10 mg tablet of methadone sometime in the week prior to presenting for bup/nx induction but he was unsure when. The long‐acting nature of methadone is the possible cause of the significant precipitated withdrawal, and is a particular point that our clinic needs to ensure we clearly educate and question our clients about prior to induction. Point of care urine drug screening is an additional tool that practitioners can utilise to reduce the risks of precipitated withdrawal, and complements thorough history taking and education of patients. It is now available at our clinic but unfortunately was not at the time of this case study.

## Discussion

In this case, the patient gave a history of heroin use, a short‐acting opioid, prior to commencing buprenorphine treatment as an outpatient, and did not disclose to treatment providers his use of methadone. Despite delaying buprenorphine doses until the patient had significant withdrawal symptoms, he experienced precipitated withdrawal—likely contributed to by long‐acting opioid use close to the initiation of buprenorphine treatment. This is unusual to have the combination of raised COWS as well as recent long‐acting opioid use. In drug and alcohol settings, the unexpected does occur and we have demonstrated that clinicians can safely manage precipitated withdrawal using bup/nx.

In our experience, it has been difficult to convince patients to continue buprenorphine or to go to the hospital for management of their withdrawal symptoms if they experience precipitated withdrawal. Our case demonstrates the importance of a good therapeutic alliance that can assist a patient to undertake rapid buprenorphine increases to overcome precipitated withdrawal, and patients can be reassured that this will alleviate their symptoms. This should take place in an inpatient setting so their symptoms can be monitored and additional doses of buprenorphine given, as well as symptomatic medications as needed. We found that repeating doses of 8 mg, with close monitoring of changes in withdrawal symptoms and for sedation, allowed us to rapidly and safely relieve our patient's symptoms. Within 24–48 h, symptoms settle and treatment continued in the community. More research is required to determine an evidence‐base for this rapid dose escalation in the setting of precipitated withdrawal, so prescribers can confidently treat this complication of buprenorphine treatment.

Patient consent to publish this case report has been provided. A document showing the patient's written consent to this publication is available on request.

## Conflicts of Interest

None of the authors have any connections with the tobacco, alcohol or gaming industry. HW has received funding for consultancies and/or expert advisory panels with Indivior, Lundbeck, Mundipharma and Pfizer. NL has received funding for research studies, consultancies and/or expert advisory panels with Indivior, Braeburn and Mundipharma. All other authors have nothing to declare.
